# Synergistic anti-cancer effects of immunotoxin and cyclosporin *in vitro* and *in vivo*

**DOI:** 10.1038/sj.bjc.6605312

**Published:** 2009-09-22

**Authors:** Y Andersson, O Engebraaten, Ø Fodstad

**Affiliations:** 1 Department of Tumor Biology, Institute for Cancer Research, Norwegian Radium Hospital, Rikshospitalet University Hospital, Oslo N-0310, Norway; 2 Faculty Division, The Norwegian Radium Hospital, University in Oslo, Oslo N-0310, Norway

**Keywords:** immunotoxin, apoptosis, cyclosporin, breast cancer, metastasis model

## Abstract

**Background:**

The clinical use of immunotoxins (ITs) has been hampered by hepatotoxicity, and the induction of a strong human-anti-IT response. The human-anti-IT response results in neutralisation of the immunoconjugates, rendering repetitive treatment inefficacious.

**Methods:**

We evaluated the combination of cyclosporin A (CsA) with various Pseudomonas exotoxin A-based ITs in human breast, cervical, and prostate cancer cell lines measured by protein synthesis, cell viability, and TUNEL assay. Furthermore, expression of essential proteins were analysed by western blot. We used cervical cancer model in nude rats to evaluate the anti-metastatic effect of the combination. The anti-immunogenic response by the CsA treatment was investigated in immunocompetent rats.

**Results:**

The combination of CsA with ITs caused remarkable synergistic cytotoxicity, in several cancer cell lines, characterised by protein synthesis inhibition, decreased cell viability, and an increased apoptotic index. Furthermore, the combination strongly inhibited formation of metastases in a cervical cancer model in nude rats with a statistically significant increase in median survival time of the combination-treated animals, as compared with those receiving a suboptimal dose of IT alone. Notably, we found in immunocompetent rats that the anti-IT immunoresponse elicited by repeated administration of IT was efficiently abrogated by CsA; notably the antibody responds towards the highly immunogenic PE was shown to be prevented.

**Conclusion:**

The combination of ITs and CsA might constitute a significant improvement in the clinical potential of systemic IT treatment of cancer patients.

Immunotoxins (ITs) represent a promising group of targeted therapeutics for cancer patients in which standard treatment is no longer an option. ITs are dimeric proteins consisting of a targeting moiety (often an antibody) covalently linked to a toxin (Ricin, Pseudomonas exotoxin A (PE), Diphtheria toxin), which by inducing arrest of protein synthesis and induction of apoptosis result in subsequent cell death (Kreitman, 2003). The targeting moiety binds to antigens expressed on cancer cells, theoretically leaving normal cells unaffected.

We have shown earlier that the 425.3PE and BM7PE increased symptom-free survival in human breast and cervical cancer models in nude rats (Engebraaten *et al*, 2000). These ITs consist, respectively, of the 425.3 (anti-EGFr) and the BM7 (anti-mucin-1) intact murine monoclonal antibody, covalently linked to the bacterial toxin PE.

The clinical use of ITs has been hampered by hepatotoxicity, neurotoxicity, vascular leak syndrome (VLS), and the induction of a strong human-anti-mouse-antibody (HAMA) response. The HAMA response, or more precisely the anti-IT antibody response, results in neutralisation of the immunoconjugate, rendering the treatment inefficacious, thus limiting the efficacy of repeated administration of the treatment. Such repeated IT administration might be needed for an efficient anti-cancer effect of IT.

Cyclosporin A (CsA) is a cyclic undecapeptide, routinely used in autoimmune disorders and organ transplantations. The CsA molecule is able to bind to both cyclophilin and calcineurin. The cyclophilins are important in protein folding, cytoplasmic trafficking of proteins, and in import of newly synthesised proteins to the mitochondria, and CsA might impede these effects by binding to cyclophilins. Calcineurin, a Ca^2+^- and calmodulin-dependent phosphatase, is a critical component of the immunosuppression pathway by transcriptionally blocking many genes involved in the establishment of the immune response of T lymphocytes and by binding to the CsA–cyclophilin complex (Clipstone and Crabtree, 1992). CsA has been demonstrated *in vitro* to be either a pro-apoptotic or an anti-apoptotic agent, depending mainly on the cell type studied and on the CsA concentration used.

Here, we document that CsA abrogates the IT-evoked, anti-IT antibody response in immunocompetent animals and hence should allow repeated administration of effective IT doses in the clinic. In parallel, the combination exerted strong synergistic effects *in vitro*, and in a nude rat model for cervical cancer it significantly inhibited the formation of metastases.

## Material and methods

### Material

The anti-EGF receptor antibody 425.3 was a gift from Dr R Reisfeld (Scripps Institute, La Jolla, CA, USA) (Mueller *et al*, 1991). The MOC31 antibody (MCA Development, Groningen, the Netherlands) detects the epithelial glycoprotein EGP-2. The BM7 antibody, which recognises a glycosylated epitope on the MUC1 mucin, was kindly provided by Dr S Kaul (Frauenklinik, Heidelberg, Germany). The antibody was conjugated to PE (obtained from Dr Darrel Galloway (University of Ohio, Columbus, OH, USA) by a thioether bond formed with the reagent sulfo-SMCC (sulfo-succinimidyl-4-(*N*-maleimidomethyl) cyclohexane-1-carboxylate) (Pierce, Rockford, IL, USA) as described earlier (Godal *et al*, 1992).

CsA for *in vitro* use was purchased from Calbiochem (San Diego, CA, USA) and was resuspended in dimethyl sulfoxide (Sigma Chemical Co, St Louis, MO, USA). Sandimmun neoral (CsA) for *in vivo* administration was from Novartis (Oslo, Norge). Sirolimus, tacrolimus, and cycloheximide (CHX) from Sigma Chemical. Ricin was a kind gift from Sjur Olsnes (Department of Biochemistry, Institute for Cancer Research, in our institution).

### Cell culture

Establishment and characterisation of the MA11 breast cancer cell line has been described earlier (Rye *et al*, 1996; Micci *et al*, 2001). The MT-1 cell line was originally obtained (Engebraaten *et al*, 2000), but has recently been determined to be a variant HeLa cell line, thereafter named HeLa^*^. The prostate cancer cell line DU-145 was purchased from American Type Tissue Culture Collection (ATCC, Rockville, MD, USA). The cell lines were grown in RPMI 1640 medium supplemented with 10% heat inactivated fetal calf serum (FCS) and glutamax (Life Technologies, Paisley, UK) and kept in a standard tissue culture incubator at 37°C. The glioblastoma cell lines U-87MG (obtained from ATCC), U-251MG, and D-54MG were grown as recently described (Hjortland *et al*, 2003). Confluent cells were detached with 0.01 M EDTA. For injecting into the animals, cells in exponential growth phase were used.

### Measurement of protein synthesis inhibition

Protein synthesis inhibition caused by IT or the protein synthesis inhibitors CHX and ricin were measured by using the [^3^H]-leucine incorporation assay (Sandvig and Olsnes, 1982). Cells (4 × 10^4^ per well) were seeded in 48-well plates and allowed to grow overnight before addition of different concentrations of IT. After 20 h incubation, the cells were washed twice with cold phosphate-buffered saline (PBS), 0.1% FCS, and incubated with [^3^H]-leucine (2 *μ*Ci ml^−1^) in leucine-free medium for 45 min at 37°C. The cells were then washed with 5% trichloroacetic acid for 10 and 5 min, respectively, and dissolved in 0.1 M KOH for at least 5 min. The resultant solution was transferred to the liquid scintillator Aquasafe 300 Plus (Zinsser Analytic, Frankfurt/Main, Germany). Sample counts were determined in a liquid scintillation counter (LKB Wallac, Perkin Elmer, Boston, MA, USA). Assays were performed in duplicate, and repeated at least three times. CsA was added 1 h before the IT. Serum (0, 0.025, 0.25, or 2.5%) was added just before addition of IT.

### Evaluation of the effect of IT on cancer cell viability

The IT effect on cell viability was measured using the CellTiter 96 Aqueous One Solution (MTS-assay) (Promega, Madison, WI, USA). Cells were seeded in 96-well plates at 3000–8000 cells/well and grown to about 40–80% confluence (depending on incubation time with IT), and the old medium was replaced with new medium containing IT and incubated at 37°C for 24 h–6 days. CsA, tacrolimus, and sirolimus were added 1 h before the IT. Serum (0, 0.025, 0.25, or 2.5%) was added just before addition of IT.

The CellTiter 96 Aqueous One Solution was then added to the wells, and the absorbance was measured 2–4 h later at a wavelength of 490 nm. The values for total viability of the treated cells were compared with the values generated for the untreated control cells and reported as the percentage cell viability. The assays were performed in triplicate, and repeated at least three times.

The results from the cell viability assay were analysed by use of CalcuSyn (version 1.1 Biosoft, Cambridge, UK), a commercially available software package, a program based on the method of Chou and Talalay (1984), which performs single and multiple drug dose–effect calculations and determines the presence of antagonism, additivity, or synergism between treatment groups on the effect of the growth inhibition caused by the drugs alone relative to the effect produced by the combination. The program returns combination index (CI) values; values < 0.9 indicate synergy, values between 0.9 and 1.1 indicate additive effects, and values >1.1 indicate antagonistic interactions.

### Western blot analysis

After IT incubation for the indicated time period, both adherent and floating cells were lysed by an SDS-boiling method. Cell pellets were resuspended in the lysate buffer (2% SDS, 1 mM Na_3_VO_4_, and 10 mM Tris–Cl (pH 7.6)), which was held at 100°C when added, and the lysates were boiled for 5 min. After six passages through a 20 × G-gauge syringe on ice, the lysates were cleared by centrifugation. Protein concentrations were then determined using the BCA protein assay (Pierce). The lysates were snap frozen in liquid N_2_ and kept at −70°C.

A portion (20 *μ*g of protein) of each lysate was fractionated by 8–12% SDS–polyacrylamide gel electrophoresis and transferred to polyvinylidene difluoride membrane (Bio-Rad, Hercules, CA, USA) by electroblotting. The filters were probed with a designated primary antibody. The anti-caspase-3 was purchased from R&D System (Minneapolis, MN, USA) and the anti-*α*-tubulin from Oncogene Research Products (San Diego, CA, USA). The anti-PARP antibody was purchased from Roche Diagnostics (Mannheim, Germany) and the anti-Mcl-1 antibody from Santa Cruz Biotechnology (Santa Cruz, CA, USA). Immune complexes were detected with appropriate HRP-coupled secondary antibodies. Peroxidase activity was visualised with enzyme-linked chemiluminescence (Amersham Pharmacia Biotech, Buckinghamshire, UK) and quantified by densitometry (Imagemaster, Amersham Pharmacia Biotech). All western blots were stained with amidoblack and probed with anti-*α*-tubulin to confirm equal loading and transfer of samples.

### TUNEL staining for flow cytometry

DNA fragmentation was quantified by terminal deoxynucleotidyl transferase-mediated dUTP-biotin nick end labelling (TUNEL). After IT incubation for the indicated time period, both adherent and floating cells were collected, washed in PBS, and fixed in 100% methanol and stored at −20°C. The terminal transferase assay kit (Boehringer, Mannheim, Germany) was used as described earlier (Lund *et al*, 2005) to detect free 3′-OH ends of cleaved DNA. Fragmented cells and debris were excluded from measurements by gating the remaining intact cells in a forward and side scatter analysis. The FACStar flow cytometer (Becton Dickinson, San Jose, CA, USA) was set up to measure forward and side scatter and FITC fluorescence (520–550 nm). FACS analysis was carried out on 1 × 10^4^ cells. The CsA was added to the cells 1 h before addition of IT at a final concentration (0.2–2 *μ*M) as indicated.

### Animals

All procedures and experiments that involved animals were approved by The National Animal Research Authority and carried out according to the European Convention for the Protection of Vertebrates used for Scientific Purposes. Nude rats (Han: rnu/rnu Rowett) and immunocompetent rats (Rowett nu/+) were bred in our rodent facility. Animals were kept in a specific pathogen-free environment, in positive pressure rooms with filtered and humidified air. The animals were kept under standard conditions and fed with standard pellets and fresh water.

### Tumour cell inoculation: experimental endpoints

Four- to five-week-old nude rats of both sexes were used. Rats injected with cells in the left cardiac ventricle (LV) were anaesthetised by s.c. injection of a mixture containing 0.1 mg kg^−1^ fentanyl, 5 mg kg^−1^ fluanison, and 2.5 mg kg^−1^ midazolam. Briefly, a microinfusion set containing a 27-gauge needle connected to an infusion tube was used. The needle was inserted through the third intercostals space into the LV of the heart after a midline skin incision. When, on aspiration, light red pulsating blood was observed in the tube, 0.2 ml of cell suspension containing 10^6^ HeLa^*^ cells or 2.5 × 10^6^ MA11 cells were injected into the ventricle, the needle withdrawn, and the skin closed with staples. The sandimmun treatment was performed in animals anaesthetised with halothane and N_2_O mixed with O_2_ and was administered approximately 6 h after cancer cell injection and thereafter everyday for a total of 5 days. The IT treatment was administered after 24 h diluted to a 100 *μ*l volume. The animals were then inspected daily with respect to symptoms of tumour-related disease and general condition. A total of 54 animals and 45 animals were injected with cancer cells, HeLa^*^, and MA11 cells, respectively. The animals were divided into four groups: control, IT, IT+CsA, and CsA alone, at least 10 animals were included per group. The animals were killed when symptoms of metastatic disease appeared. As described previously, disease related to growth of HeLa^*^ or MA11-derived metastases caused neurological symptoms, cachexia, and weight loss (Engebraaten *et al*, 2000). The time from inoculation of the tumour cells to the appearance of symptoms of metastasis was recorded as the mean latency time. The Kaplan–Meier plot estimated the survival function from the life-time data. The log-rank test was used to assess the significance of observed differences in survival times between the different treatment and control groups.

### Anti-IgG IT in immunocompetent rats

Immunocompetent rats (Rowett nu/+) weighing approximately 400–500 g were used in this study. The animals were kept under standard conditions and fed with standard pellets and fresh water. The rats were divided into four groups: control (*n*=7), IT (*n*=10), IT+CsA (*n*=10), and CsA (*n*=7). The treatment schedule of the immunocompetent rats started at day 0 with CsA (10 mg kg^−1^ per day for 5 days) injected intravenously (i.v.) and then every day for a total of 5 days in a volume of 100 *μ*l, in the CsA-treated animals. The control group received PBS at day 0 and then every day for a total of 5 days in a volume of 100 *μ*l. A single i.v. injection of MOC31PE (100 *μ*g kg^−1^) was given at day 1 to the two groups, IT and IT+CsA. The treatment schedule was repeated every second week for a total of 8 weeks. Blood samples were collected at day 0 before the start of the experiment and then every second week. The physical condition of the animals was regularly monitored. No toxicity was seen when monitoring animals for loss of body weight and decline in general condition. Blood was drawn from the rats and allowed to clot overnight at 4°C. The blood was then centrifuged at 2800 r.p.m. for 10 min at 4°C and the serum layer was removed, aliquoted, and stored in at 4°C. Ninety-six-well Costar ELISA plates were coated with MOC31PE and analysed for anti-IgG IT. The anti-IT IgG was measured by ELISA and plotted against a standard curve.

## Results

### CsA synergistically increases the IT-mediated cell death in MA11 human breast cancer cells

**Figure 1:**
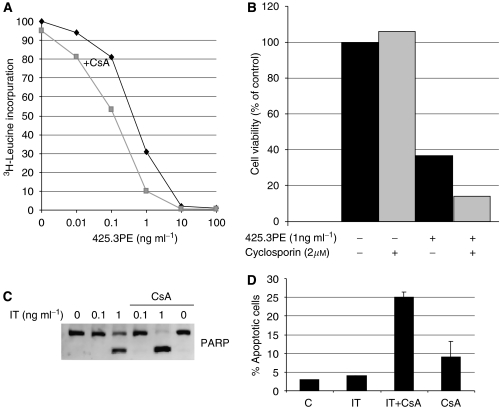
CsA enhanced the cytotoxicity of the 425.3PE immunotoxin in MA11 breast cancer cells. (**A**) Cells were incubated with different concentrations of 425.3PE and/or a fixed dose of CsA (2 *μ*M) for 20 h. The rate of protein synthesis in each cell culture was expressed as a percentage of the value obtained in untreated control cells. The data are expressed as the mean of triplicates, representative of two independent experiments. (**B**) Cells were incubated with 1 ng ml^−1^ IT 425.3PE and/or CsA (2 *μ*M) for 20 h before the MTS assay was performed. The results represent the means of four determinations and the assay was repeated at least three times. (**C**) After treatment with 425.3PE and/ or a dose of CsA (2 *μ*M) for 5 h, MA11 cell lysates were subjected to western blotting. The filters were incubated with anti-PARP antibody as described in Material and Methods. The gels represent at least three independent experiments. Anti-*α*-tubulin detection was used to confirm equal loading of proteins (data not shown). (**D**) MA11 cells exposed to 425.3PE (0.1 ng ml^−1^) for 4 days and pretreated or not with CsA added 1 h before the IT at a final concentration of 2 *μ*M. The numbers represent percentage of apoptotic cells detected by TUNEL staining of treated and untreated cells analysed by flow cytometry. The experiments were performed twice.

The concentration-dependent efficacy of the combination of 425.3PE and CsA on protein synthesis was examined in MA11 breast cancer cells. The IC_50_ value for inhibition of protein synthesis was 1 ng ml^−1^ of 425.3PE alone compared with approximately 0.1 ng ml^−1^ of 425.3PE combined with CsA (2 *μ*M) (Figure 1A), whereas CsA alone only gave a marginal effect. These results were supported by the findings that the combination with CsA (2 *μ*M) effectively increased the anti-cancer effect of 425.3PE (1 ng ml^−1^) measured in the MTS assay. Thus, after 24 h the cell viability decreased by more than 50%, from 38% to 17%, compared with untreated cells (Figure 1B), whereas CsA alone had no effect. In the concentrations used, neither the PE toxin nor the 425.3 antibody alone, or in combination with CsA, had any effects on protein synthesis and cell viability (not shown), showing that the synergistic cytotoxicity of IT and CsA is a specific event.

We have shown earlier that our PE-based ITs induce apoptosis in several types of cancer cells (Andersson *et al*, 2004, 2006). Hence, the synergistic effect of the IT and CsA combination could conceivably be mediated through induction of apoptosis. Western blot analysis showed an efficient cleavage of PARP (poly (ADP-ribose) polymerase) to the 85 kDa inactive fragment in MA11 cells treated with 425.3PE (1 ng ml^−1^)+CsA (2 *μ*M) for 5 h compared with the cells treated with 425.3PE only (Figure 1C). CsA alone had no influence on PARP inactivation. In the MA11 breast cancer cells treated with a sub-toxic dose of 425.3PE (0.1 ng ml^−1^) for 4 days with or without CsA (2 *μ*M) (Figure 1D), the apoptotic index after treatment was measured by TUNEL analysis. In the combination-treated cells, the apoptotic index was 25% compared with 5% in the IT-only treated cells, 10% in CsA-treated cells, and 3% in untreated cells. The observation suggests that induction of apoptosis may be the major contributing factor to the synergistic anti-cancer properties of the combination therapy.

### Strong synergistic effects of the IT and CsA combination in cancer cells of different origins

The potential of the combination treatment of IT+CsA was further investigated in several different cancer cell lines (cervical; HeLa^*^, prostate; DU145 and glioblastomas; U87 MG and D54 MG (data not shown)), and also using different ITs (BM7PE, MOC31PE, and 425.3PE).

**Figure 2:**
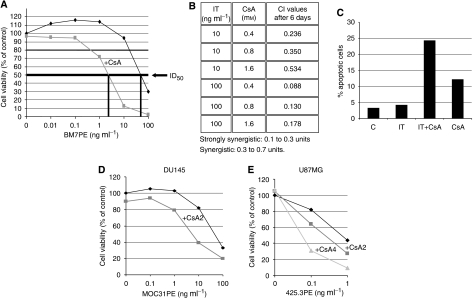
CsA synergistically enhanced the IT effect in several types of cancer cells (**A**) HeLa^*^ cells were incubated with different concentrations of the BM7PE IT and/or a dose of CsA (2 *μ*M) for 6 days before the MTS assay was performed. (**B**) The table shows the calculated combination index (CI) determined by Calcusyn software (version 1.1, Biosoft). Data are expressed as the mean of triplicates, representative of two independent experiments. (**C**) HeLa^*^ cells exposed to BM7PE (1 ng ml^−1^) for 6 days and pretreated or not with CsA added 1 h before the IT at a final concentration of 2 *μ*M. The numbers represent percentage of apoptotic cells detected by TUNEL staining of treated and untreated cells analysed by flow cytometry. The experiments were performed twice. (**D**) DU145 prostate cancer cells were incubated with different concentrations of the MOC31PE as indicated in the figure and/or a dose of CsA2 (2 *μ*M) for 3 days before the MTS assay was performed. The data are expressed as the mean of triplicates, representative of two independent experiments. (**E**) U87MG glioblastoma cells were incubated with different concentrations of 425.3PE as indicated in the figure and/or a dose of CsA2 (2 *μ*M) or CsA4 (4 *μ*M) for 3 days before the MTS assay was performed. The data are expressed as the mean of triplicates, representative of two independent experiments.

HeLa^*^ cells were incubated with increasing doses of BM7PE (0.01–100 ng ml^−1^) alone and with increasing concentration of CsA (0.2–2 *μ*M) for 6 days (data shown only for 2 *μ*M of CsA) (Figure 2A). The rationale to use HeLa^*^ cells and BM7PE in this study was the results in our previous work in the HeLa^*^ metastasis model, in which the BM7PE alone showed only a limited anti-metastatic effect (Engebraaten *et al*, 2000). Our objective was to examine if the combination therapy could improve the anti-metastatic effect of BM7PE. *In vitro* the cell viability of HeLa^*^ decreased with increasing doses BM7PE alone and when combined with CsA the increase in cell death was synergistically enhanced, resulted in approximately 40-fold lower IC_50_ compared with IT monotherapy (Figure 2A). Very low BM7PE doses alone, equal to or less than 1 ng ml^−1^, resulted in slightly increased cell viability, suggesting induction of pro-survival signals at these concentration level (Andersson *et al*, 2006). However, even at these low IT doses (BM7PE ⩽1 ng ml^−1^) we found that, the combination resulted in a synergistic increase in cell kill; IT (1 ng ml^−1^) plus CsA (2 *μ*M) decreased the cell survival by 40% compared with IT alone (114%), whereas CsA (2 *μ*M) mono-treatment showed no effect.

The Calcusyn program was used to calculate the CI values for BM7PE (0.01, 0.1, 1.0, 10, and 100 ng ml^−1^) plus CsA (0.2, 0.4, 0.8, 1.6, and 2 *μ*M). CI values in the order of 0.1–0.3 units are considered ‘strongly synergistic’, and ‘synergistic’ ranges lie between 0.3 and 0.7 units. Synergistic or strongly synergistic effects (CI values between 0.1 and 0.7 units) were obtained with 10 and 100 ng ml^−1^ of IT in combination with CsA (0.4, 0.8, and 1.6 *μ*M) (Figure 2B). The combination was determined to be antagonistic at the lowest IT concentrations (0.01–1 ng ml^−1^), independent of CsA concentrations with CI values over 1 (data not shown). Thus, IT and CsA interact synergistically only at concentrations where IT alone caused cell death. ‘High’ concentrations of CsA alone, 0.8, 1.6, and 2 M, gave up to more than 40% reduction in cell viability in HeLa^*^ cells (data not shown), whereas lower CsA concentrations (0.2 and 0.4 *μ*M) resulted only in a minor effect on cell viability on 6 days of treatment.

HeLa^*^cells treated with BM7PE+CsA (1 ng ml^−1^+2 *μ*M) were analysed for TUNEL-positive cells (Figure 2C) and the results showed that the observed synergistic effect in cell viability was at least in part because of an increase in apoptotic cells. Thus, after 6 days of treatment HeLa^*^ cells showed 4% (IT) and 24% (combination) positive cells compared with 3% for untreated cells and 12% for CsA-only treated cells.

DU145 prostate cancer cells were treated with MOC31PE (0.1, 1.0, 10, and 100 ng ml^−1^) with or without CsA (2 *μ*M) (Figure 2D). The IT (10 ng ml^−1^)+CsA combination decreased the cell viability by 60% compared with 20% by IT (10 ng ml^−1^) and 10% by CsA alone, all compared with untreated control cells. Compared with the other cell lines, U87MG glioblastoma cells were less sensitive to the CsA treatment. Thus, the CsA concentration had to be increased to achieve a synergistic combination-induced cell death (Figure 2E). In these cells, the combination of 425.3PE (0.1 ng ml^−1^) and CsA (4 *μ*M) caused a synergistic decrease in cell viability (70% cell kill), IT alone 20%, whereas CsA alone had only a minor effect on cell viability. Similar results were obtained for the glioblastoma cell line D54MG (data not shown).

### The immunosuppressors tacrolimus and sirolimus had no beneficial effect on IT-induced cell death

**Figure 3:**
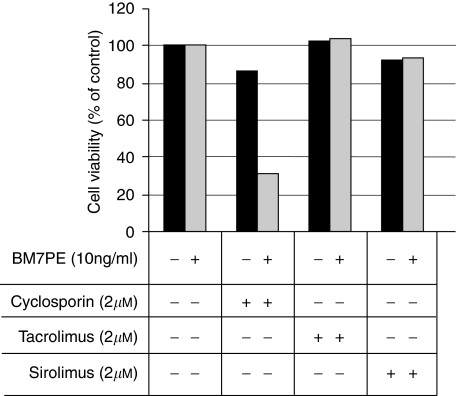
Tacrolimus or sirolimus had no additive or synergistic effect on IT-induced cell death. The cervical cancer cell line HeLa^*^ cells were incubated with a fix dose of BM7PE (10 ng ml^−1^) and/or a dose (2 *μ*M) of CsA, tacrolimus, and sirolimus for 3 days before the MTS assay was performed. The data are expressed as the mean of triplicates, representative of two independent experiments.

Two widely used immunosuppressors, tacrolimus (FK-506) and sirolimus (rapamycin), were tested in combination with IT. HeLa^*^ cells were incubated with BM7PE (10 ng ml^−1^) in combination with either CsA (2 *μ*M), tacrolimus (2 *μ*M), or sirolimus (2 *μ*M) for 3 days before the MTS assay was performed. Interestingly, the immunosuppressors tacrolimus and sirolimus (0.2–8 *μ*M) did not influence the IT-induced cell death, in contrast to the benefits of the synergistic effect observed with CsA (Figure 3). Furthermore, the immunosuppressors did not affect the IT-induced cell death even at concentration up to 8 *μ*M (data not shown). No synergism was observed with tacrolimus and sirolimus when the same experiments were performed with MA11 and glioblastoma D54MG cells, but sirolimus was by itself highly cytotoxic to MA11 cells (data not shown). The data suggest that the synergistic CsA-mediated IT-induced reduction in cell viability is most likely unrelated to the mechanisms that CsA, tacrolimus, and sirolimus have in common by selectively blocking transcriptional activation of defence-related genes early in the T-cell signal transduction pathway. However, the difference might be explained by the fact that the drugs have different cellular targets.

### Combination induced increased animal survival in metastasis models in nude rats

**Figure 4:**
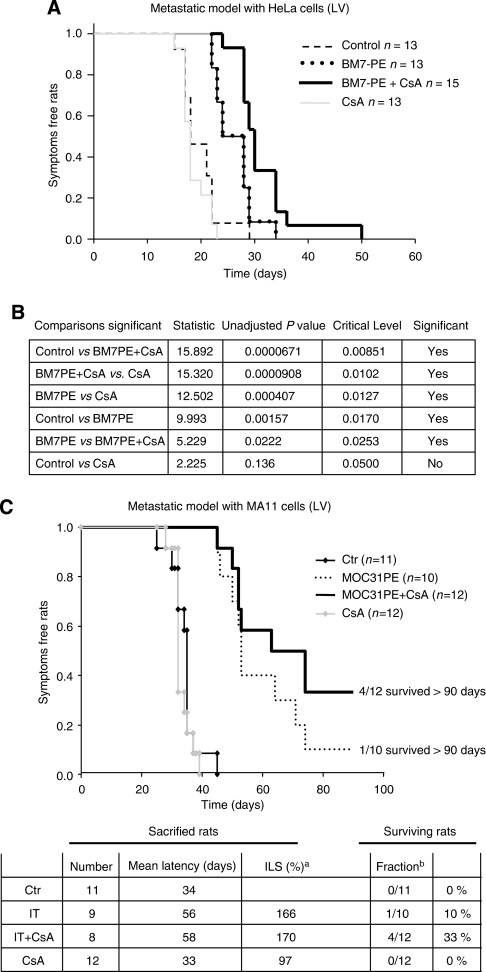
Survival curves (**A**) of nude rats injected with HeLa^*^ cells in left cardiac ventricle (LV). Nude rats were treated with IT and/or CsA or control vehicle (PBS) in a volume of 100 *μ*l. CsA (10 mg kg^−1^ per day) was given every day for 5 days starting on day 0 (cell injection, 1 × 10^6^ cells), and BM7PE (10 *μ*g) was given as a single dose on day 1 after tumour cell injection. (**B**) A statistically significant prolongation in survival (*P*=0.022) of rats in the IT and CsA combination-treated group compared with mono-IT therapy. The results were calculated by the all pair-wise multiple comparison procedure by the Holm–Sidak method. (**C**) The combination of IT MOC31PE and CsA increased survival of nude rats injected with MA11 cells in LV. The rats were treated with a single injection of IT MOC31PE (10 *μ*g) and/or CsA as outlined in (**A**). ^a^ILS, increase in lifespan (median survival time-treated group/median survival time control group−1) × 100%. Survivors (survivors/total number of animal per group). ^b^Follow-up period >90 days. CsA (CsA, sandimmun, Novartis).

The observed advantageous *in vitro* effects of the combination of IT and CsA were tested in two of our previously reported human tumour models in immunodeficient rats, simulating micrometastatic disease. The rats were injected with either HeLa^*^ cells or MA11 cells in the LV, and 6 h later the animals were treated i.v. with 10 mg per day of CsA daily for 5 days. BM7PE was given i.v. on day 1 after HeLa^*^ cell injection as a single bolus (10 *μ*g per 60 g animal) and MOC31PE (10 *μ*g per 60 g animal) was given i.v. on day 1 after MA11 cells injection. In untreated animals, the metastatic disease caused debilitating symptoms. In the cervical tumour model, the combination (*n*=15) was more effective in inhibiting the development of metastasis compared with IT monotherapy (*n*=13), as demonstrated in two independent experiments (four treatment groups, total 13–15 animals/treatment group) (Figure 4A). Notably, a statistically significant prolongation in survival (*P*=0.022) was found (Figure 4B), as calculated by the All Pairwise Multiple Comparison Procedures using the Holm–Sidak method. No toxicity was observed with this CsA dose or even by the double dose of CsA (20 mg per day) in combination with IT (10 *μ*g per 60 g animal). The same experimental setting was used for a breast cancer model (MA11 cells) in rats treated with the MOC31PE. A trend towards a positive effect of the combination was obtained, but statistical significance was not reached because of one long-term survival in the IT group (*n*=10). The mean latency in days was nearly the same for the IT and the IT+CsA group, 56–58 days. Importantly, the surviving fraction of rats in the combination group was higher compared with IT mono-therapy group; in the IT group 1 out of 10 rats survived more than 90 days compared with 4 out of 12 rats in the combination group. However, the CsA alone had no significant inhibitory effect on metastasis and none of the agents alone or in combination caused any weight loss.

### CsA prevented antibody immune response against IT *in vivo*

**Figure 5:**
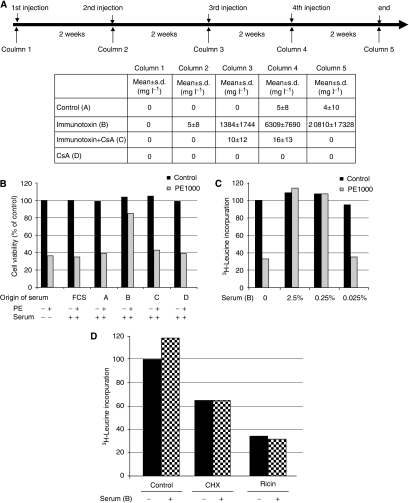
Immunotoxin (IT)-induced antibody generation in immunocompetent rats was suppressed by CsA. The upper part of the (**A**) shows the scheduling of the treatment. Treatment with MOC31PE leads to the development of anti-IT IgG in immunocompetent rats, as analysed by ELISA (**A**, lower part). The table shows the anti-IT IgG levels in the rat sera. Day 0 (column 1) blood was collected from all animals and analysed as a zero reference of anti-IT IgG. The rats in groups C and D were treated with CsA only, 10 mg kg^−1^ per day for 5 days (days 0–4). On day 1 the animals in group B and C were injected i.v. with MOC31PE (100 *μ*g kg^−1^). Treatment repeated as outlined every second week, for a total of 8 weeks, induced a massive generation of anti-IT IgG in group B, which was completely prevented in rats given MOC31PE and CsA (group C). The untreated and CsA groups (A and D) did not develop antibodies. *n*=10 per group for the IT and IT+CsA groups, and *n*=7 for the control (vehicle) and CsA alone groups. (**B**) Serum from IT-treated immunocompetent rats inhibited the Pseudomonas Exotoxin A (PE)-induced cytotoxicity. Glioblastoma cells U251 MG with a high expression of PE receptors (*α*-macroglobulin receptor) were incubated with PE (1000 ng ml^−1^) with or without the presence of 2.5% serum from the treated rats, groups A–D column 5, or fetal calf serum (FCS) for 24 h before the MTS assay was performed. The data are expressed as the mean of triplicates, representative of two independent experiments and representative of sera from five animals per treatment group. (**C**) Serum from IT-treated animals blocked PE-induced inhibition of protein synthesis. Glioblastoma cells U251MG were incubated with PE (1000 ng ml^−1^) with or without the presence of serum (0.025% or 0.25% or 2.5%) from the IT-treated animals (group B column 5) for 24 h before the protein synthesis assay was performed. The data are expressed as the mean of triplicates, representative of two independent experiments. The experiment was repeated with four different IT-treated animal sera showing similar results. (**D**) Serum from IT-treated immunocompetent rats did not block the effects of other protein synthesis inhibitors. Glioblastoma cells U251MG were incubated with ricin (1 *μ*M) and cycloheximide (CHX) (5 *μ*g ml^−1^) with or without the presence of serum (2.5%) from the IT-treated animals (group B column 5) for 24 h before the protein synthesis assay was performed. The data are expressed as the mean of triplicates, representative of two independent experiments. The experiment was repeated with two different IT-treated animals serum (group B column 5) showing similar results.

The possibility that CsA could inhibit IT-induced antibody response was studied in immunocompetent rats. The animals were treated with CsA, 10 mg kg^−1^ per day one to five, with bolus injections of IT (100 *μ*g kg^−1^) given at day 2 in the 2-weeks cycle. A total of four treatment cycles were given. Serum from IT-treated animals was analysed for anti-IT antibodies in an ELISA assay. Two weeks after the second injection of IT, anti-IT IgG was already highly elevated (1384±1744 *μ*g l^−1^) in IT-treated animals (*n*=10) (Figure 5A). During the sequential administration of IT the anti-IT levels continued to increase dramatically, and 2 weeks after the fourth injection of IT (*n*=10) the mean total anti-IT antibody levels had reached 20 810±17 328 *μ*g l^−1^, after a treatment period of 8 weeks. Notably, no anti-IT antibodies were detected in the combination-treated group (IT+CsA, *n*=10) with mean plasma anti-IT level close to the background (10±12 *μ*g l^−1^). The plasma anti-IT IgG in control mice (untreated or CsA alone, *n*=7 per group) was undetectable, as expected. None of the agents alone or in combination caused any weight loss.

The specificity of the induced antibody response was examined in U251MG glioblastoma cells, which has a high expression of the *α*-macroglobulin receptor (Hussaini *et al*, 1999), which is the receptor protein to which the PE binds. Serum from IT-treated immunocompetent animals was analysed for the presence of neutralising anti-IT-antibodies on PE-induced cytotoxicity in the MTS assay (Figure 5B) and on PE-induced inhibition of protein synthesis (^3^H-leucine incorporation assay) (Figure 5C). In U251MG cells treated for 24 h with PE alone (1000 ng ml^−1^) caused a more than 60% reduction in cell viability, which was almost completely blocked by the addition of serum (2.5%) from the animal group that had been repeatedly (four times) treated with IT. This observation suggests that the anti-IT antibody response is not only evoked by the murine monoclonal antibody part of the IT but also by the PE moiety. Importantly, when serum from animals treated with the IT+CsA combination was used, no inhibition of PE-induced cell death was seen. Fetal calf serum as well as serum from the vehicle-treated and CsA-treated control groups had no effect on PE-induced cytotoxicity in U251MG cells (Figure 5B). The PE-induced inhibition of protein synthesis was also demonstrated in U251 MG cells (Figure 5C). PE alone (1000 ng ml^−1^) decreased protein synthesis to 30% of untreated control levels, whereas the addition of serum (2.5% and 0.25%) from IT-treated animals completely hindered the PE-induced inhibition of protein synthesis (0.025% serum had no effect).To exclude the possibility that components involved in protein synthesis could be unspecifically inhibited by the rat serum, U251MG glioblastoma cells were incubated with ricin (1 *μ*M) and CHX (5 *μ*g ml^−1^) in the presence and absence of IT-treated animal serum (2.5%) for 24 h before measuring the protein synthesis. The ricin-induced and the CHX-induced inhibition on protein synthesis, 70% and 35% reduction, respectively, were not affected by the addition of serum (Figure 5D).

The results clearly argue for the importance of suppressing the specific anti-IT response generated in IT-treated animals by including CsA.

## Discussion

Previously, we have studied the anti-cancer properties and intracellular mechanisms involved in 425.3PE-induced effects *in vitro* in the human breast cancer cell line MA11 (Andersson *et al*, 2004, 2006). The aim of this study was to further improve the efficacy of ITs and attempt to minimise the immunogenic antibody response to ITs, a major challenge to the *in vivo* use of ITs as they prevent the effect of repeated administration. CsA was chosen as a known potent and clinically important immunosuppressive agent.

In the experiments in MA11 cells, the combination of IT and CsA acted synergistically on protein synthesis inhibition and on cell death with increased induction of apoptosis. The DNA fragmented fraction increased more than 10-fold when a low dose of IT (0.1 ng ml^−1^), not able to induce DNA fragmentation by itself, was combined with CsA. The data show that a close to non-cytotoxic IT dose became clearly cytotoxic when used in combination with CsA.

Similar to CsA, the two immunosuppressive drugs, tacrolimus and sirolimus (rapamycin), are used clinically to prevent immunologic rejection after solid-organ transplantation. Our findings indicate that despite the similar mechanistic effects of these immunosupressor, only CsA had the ability to synergistically increase the cytotoxicity of IT *in vitro*. This difference might be explained by the fact that the drugs have different cellular targets (Arceci *et al*, 1992; Weischer *et al*, 2007).

The IT+CsA combination was examined in a number of cell lines of different cancer types: cervical HeLa^*^, breast T47D and MA11, prostate DU145, glioblastoma D54MG (all with mutated p53), and glioblastoma U87MG (with wild-type p53). Our findings clearly indicate that cancer cells mutated in p53 are, independent of cancer origin, likely to be sensitive to the combination of IT and CsA. TP53 is mutated in ∼50% of human cancers (Soussi and Wiman, 2007), and our findings are important as TP53-mutated tumours are commonly resistant to radiation and to the majority of anticancer drugs used in the clinic. Notably, the synergistic increase in IT cytotoxicity by CsA was independent of the cellular target (EGFr, EGP2, and mucin-1).

The mechanisms and the factors responsible for the synergistic effects have not yet been fully elucidated. However, one likely explanation is a direct triggering of apoptosis illustrated by increased PARP inactivation and DNA fragmentation, rather than interference with cell-cycle events as the distribution of the cells in different cell-cycle phases was not changed (data not shown). The explanation for the increased fraction of positive TUNEL-stained cells induced by CsA alone might be related to the CsA inhibitory effect on calcineurin (protein phospatase 2B), a factor known to be involved in reduced DNA repair (Weischer *et al*, 2007). In our experiments, CsA alone (0.1–2 *μ*M for 5 or 24 h depending on cell line) caused no PARP inactivation. Importantly, several *in vitro* studies have shown that CsA alone can induce apoptosis (PARP inactivation) although at much higher concentrations (30–60 *μ*M) (Pyrzynska *et al*, 2002; Ciechomska *et al*, 2005). The *in vitro* data on the combination of IT and CsA encouraged us to examine the effects *in vivo*. In a HeLa^*^ cervical cancer model and a MA11 breast cancer model in nude rats administration of a suboptimal dose of BM7PE and MOC31PE, respectively, and used in combination with CsA did reduce the metastatic capacity of the HeLa^*^ cells and MA11 cells and prolonged the survival time of the animals compared with IT alone. The HeLa^*^ cells metastasize with a shorter lag period than the MA11 cells even with a lower number cells injected, as also reported earlier (Engebraaten and Fodstad, 1999). Notably, in the MA11 tumour model, the fraction of surviving animals compared with untreated animals was much larger in the IT+CsA group (33%, *n*=12) compared with IT group (10%, *n*=10), whereas the increase in lifespan of rats developing metastases was the same for both groups, 170% compared with untreated control animals. Recently, it was shown that CsA could inhibit intracranial glioma growth *in vivo* (Sliwa *et al*, 2007) most likely because of an inhibition of the PI-3/Akt pathway (Ciechomska *et al*, 2003). In our models, CsA alone had no effect on metastasis development. Moreover, the fact that the observed synergistic effect was shown in an immune-deficient nude rat model indicates that the pro-apoptotic combination effect of IT and CsA *in vitro* and *in vivo* is not linked to the immunosuppressive activity of CsA.

Notably, in immunocompetent rats, the combination with CsA mediated an efficient block of the anti-IT antibody response, which otherwise impedes effective IT therapy. The treatment schedule of IT in these animals was translated from the ongoing phase I study of IT alone at The Norwegian Radium Hospital (unpublished) in which IT is given every second week and repeated four times. In the IT-treated group of animals, the level of anti-IT antibodies was already high after only two IT injections and increased further during the treatment period, corresponding to the levels of anti-IT antibodies found in the clinical phase I study (unpublished results). Serum from the animals strongly neutralised the cytotoxicity of PE or IT in an *in vitro* cell cytotoxic MTS assay. Importantly, when CsA was administrated together with IT no anti-IT antibody development was seen, as demonstrated by both ELISA and in the cytotoxic MTS assay. The data imply that the use of a combination of IT and CsA in the clinic has a promising potential. CsA has been previously shown to enable repeated administration of monoclonal antibody therapy in patients by reducing the HAMA response (Ledermann *et al*, 1988; Weiden *et al*, 1994). Notably, the amount of CsA administered in the *in vivo* experiments in rats was lower, based on several publications (Palomero *et al*, 2001; Lee *et al*, 2002), that the doses commonly used in patients for immunosupression, as calculated from commonly used guidelines for conversion of doses between different species (www.fda.gov/Cber/gdlns/dose.ht m). However, whether CsA can inhibit the antibody formation to the IT will be studied in a clinical trial in 2009 in our clinical trials unit.

In earlier clinical trials with ITs, the patients have aquired VLS, linked to a damage in the capillary vessel wall probably caused by (IT damage or) specific but unwanted binding of the antibody to the endothelial cells. The VLS has never occurred in our preclinical animal models (own data, unpublished).

Critical concerns of using fully PE linked to fully mouse antibody have been raised, and on this background modified PE-containing ITs have been preferred for clinical trials. However, clinical beneficial effects have not been obtained, possibly related to pharmacokinetic and scheduling factors. We have documented in a phase I study a safe administration of the full IT, which can easily be produced in the necessary amounts for clinical use. Owing to the size of the full IT molecule the most advantageous clinical setting for our approach would be patients with minimal residual disease, for example adjuvant therapy.

Critical concerns of long-term CsA usage in transplanted patients have been raised, as one serious potential side effect is an increase risk for certain cancers. However, other and contradictory results show that CsA might have a positive function in cancer prevention (Weischer *et al*, 2007). According to medical data bases, a daily CsA dose (3–7 mg kg^−1^) was used in several clinical studies for months without any significant side effects including cancer (Frei *et al*, 1998; Koo, 1998; Levy *et al*, 2002; Vakeva *et al*, 2008). Importantly, IT combination therapy with CsA will most likely only be used in an adjuvant short-term treatment in clinic, for example, CsA for 5 days and 9 days off and then the same schedule repeated for six more weeks.

In summary, we have shown a novel and effective anti-cancer combination therapy with IT and CsA both *in vitro* and in nude rats. The combination therapy might facilitate the use of IT in clinic, as the combination simultaneously increased the anti-cancer activity of IT and efficiently inhibited the generation of neutralising anti-IT antibodies, which has been preventing effective prolonged IT therapy.
